# *Robinsoniella peoriensis* infection following surgery for scoliosis: a case report

**DOI:** 10.1186/1752-1947-6-174

**Published:** 2012-06-28

**Authors:** Nadim Cassir, Laurine Laget, Aurélie Renvoisé, Jean-Marie Gennari, Michel Drancourt

**Affiliations:** 1 Unité de Recherche sur les Maladies Infectieuses et Tropicales Emergentes, UMR CNRS-6236, IRD189, Méditerranée-Infection, Aix-Marseille-Université, 27 Boulevard Jean Moulin, 13005, Marseille, France; 2 Service de Chirurgie Orthopédique, Hôpital Nord, Assistance Publique-Hôpitaux de Marseille, Chemin de Bourrely, 13915, Marseille, France; 3 Unité de Recherche sur les Maladies Infectieuses et Tropicales Emergentes, Faculté de Médecine, 27, Boulevard Jean Moulin, Marseille cedex 5, France

## Abstract

**Introduction:**

*Robinsoniella peoriensis* was recently identified as a Gram-positive, spore-forming, anaerobic bacillus originally isolated from swine manure storage pits. Seven isolates have been subsequently reported from human sources.

**Case presentation:**

We report the case of an infection caused by *R. peoriensis* in a 45-year-old Caucasian woman after posterior instrumentation correction of idiopathic thoracolumbar scoliosis. The identification was made by culture of samples inoculated onto blood agar and chocolate agar and was confirmed by 16 S ribosomal ribonucleic acid gene sequencing.

**Conclusions:**

We discuss similar cases suggesting that *R. peoriensis* is responsible for health care-associated infections with the colonic flora as a potential source of infection.

## Introduction

A microbiological survey of swine manure yielded unknown isolates of anaerobic, Gram-positive, non-motile, spore-forming, short oval to rod-shaped bacteria [1]. Six years after the isolation of these unknown Gram-positive anaerobes, a representative isolate was phenotypically and phylogenetically characterized [2]. It was determined that this isolate belonged to the phylum Firmicutes within the family Lachnospiraceae. Phylogenetic analysis showed that this organism belonged to the clostridial ribosomal ribonucleic acid (rRNA) cluster XIVa subgroup, and the closest related genus was *Ruminococcus*. The organism was named *Robinsoniella peoriensis.* Since the initial description of this organism from an environmental source, only seven isolates have been reported from human sources [3-5]. We report what is, to the best of our knowledge, the first case of neurosurgical infection caused by *R. peoriensis* and discuss similar cases suggesting that *R. peoriensis* is responsible for health care-associated infections.

## Case presentation

A 45-year-old Caucasian woman was admitted to our hospital for surgical correction of progressive right convex scoliosis. The patient had suffered from an unbalanced adolescent idiopathic thoracolumbar right scoliosis with improvement in adulthood. A pre-operative physical examination revealed painful, rigid right thoracolumbar scoliosis and unsupported sitting. A radiograph showed right convex thoracolumbar scoliosis of 68° with a type IV pelvic obliquity graded according to the classification of King *et al*. [6]. Her medical history revealed chronic urinary tract infections. She was treated with single-stage scoliosis correction involving posterior instrumentation (Euros spinal system; Euros, La Ciotat, France) from T4 to L5. The spondylodesis was completed by applying allograft bone chips (Etablissement Français du Sang Alpes-Méditerrannée, Tissue Bank, Marseille, France) over the laminae in the thoracolumbar region. Prophylactic cefazolin (1000 mg) was administered intravenously at the induction of anesthesia. At day 8 after the operation, the wound produced negligible clear fluid without any sign of infection and our patient was discharged to a rehabilitation center. At day 16 after the operation, she developed a fever (38.6°C) and was readmitted to our hospital. A physical examination found an enlarged distal wound dehiscence with increased fluid production. Remarkable laboratory parameters included an increased level of C-reactive protein (65 mg/L; normal is less than 10 mg/L), a white blood cell count of 10.9 cells×10^9^/L (normal is 4 to 10 cells×10^9^/L), and an erythrocyte sedimentation rate of 150 mm (normal is less than 8 mm). A thorough debridement of necrotic tissue and the removal of the bone grafts were performed with the instrumentation left in place. Samples were collected from various deep areas by fluid aspiration and from the applied bone graft. Therapy with three grams of amoxicillin/clavulanate daily was initiated immediately after appropriate intra-operative cultures were obtained.

Three intra-operative specimens were received in the laboratory; direct Gram staining was negative. Samples were inoculated on blood agar and chocolate agar (bioMérieux, Marcy l’Etoile, France) and incubated at 37°C both aerobically with 5% carbon dioxide and anaerobically. All specimens incubated under anaerobic atmosphere yielded positive cultures with tiny, non-hemolytic colonies of Gram-positive rod-shaped bacilli. *In vitro* susceptibility testing using the disk diffusion method was interpreted in accordance with the guidelines of the Clinical and Laboratory Standards Institute [7]. It indicated susceptibility to amoxicillin/clavulanate, rifampicin, imipenem, metronidazole, clindamycin, and vancomycin and resistance to penicillin G. An attempt was made to identify the isolate by using matrix-assisted laser desorption/ionization time-of-flight mass spectrometry (MALDI-TOF-MS) (Bruker Daltonics, Bremen, Germany). Non-inoculted alpha-cyano-hydroxycinnamic acid matrix was used as a negative control, and positive control consisted of 1.5μL of Bruker Bacterial Test Standard, a protein extract of *Escherichia coli* DH5alpha. The two negative control spots remained negative, and the two positive control spots identified *E. coli* with score values of 2.281 and 2.363, thus validating the MALDI-TOF-MS manipulation. The isolate yielded a non-identifying spectrum with identification score values of greater than 1.699. PCR amplification and sequencing yielded a 1466-base pair (bp) 16 S rRNA gene sequence differing at only 10 nucleotide positions from that of reference *R. peoriensis* (GenBank AF445285; 99.3% sequence similarity) [2]. In parallel, a 951-bp 16 S rRNA gene sequence obtained from two patients’ specimens as previously reported [8] differed at only three nucleotide positions (similarity of 99.6%) from that of *R. peoriensis* reference (GenBank AF445285). Amoxicillin/clavulanate was replaced by a combination of rifampicin (300 mg) and clindamycin (600 mg) administered orally at eight-hour intervals for six months. Our patient was discharged five days after surgery. Wound healing and temperature were monitored at regular intervals at the out-patient clinic. At three-month follow-up, our patient was afebrile, the wound had healed, and there was no longer biological inflammation. At one-year follow-up, radiographs showed a complete correction of the scoliotic deformity and no instrumentation failure.

## Discussion

We report the first *R. peoriensis* isolate made in our laboratory. We interpreted this isolate as being responsible for the orthopedic device infection in our patient, as it was isolated in pure culture from three different surgical specimens collected at the time our patient had clinical, radiological, and biological evidence of local infection. Also, an *R. peoriensis*-specific 16 S rRNA gene sequence was detected directly in two specimens. We observed that MALDI-TOF-MS failed to identify the isolate, as *R. peoriensis* profile was not included in its database. This reinforces the usefulness of broad-range PCR for the diagnosis of osteoarticular infections, particularly in case culture-negative infection in which fastidious organisms may be involved [8]. Likewise, the accuracy of 16 S rRNA gene sequencing for the identification of anaerobic organisms from blood cultures was demonstrated recently [9].

Our patient was cured after the administration of an appropriate antibiotic therapy guided by the results of the *in vitro* antibiotic susceptibility testing of the isolate. Previous susceptibility testing of four human isolates showed resistance to penicillin (minimal inhibitory concentration, or MIC, of 6 to 8 g/mL) and clindamycin (MIC of 8 to 12 g/mL) but susceptibility to piperacillin-tazobactam (MICs of 8/4 to 16/4 g/mL) and metronidazole (MICs of 0.75 to 1.0 g/mL) [4]. The authors noted that although the isolate from patient three was never tested for antimicrobial susceptibility, the use of clindamycin in this case could have contributed to treatment failure (Table 1). Another isolate was susceptible to metronidazole, cefoxitin, imipenem, amoxicillin-clavulanic acid, and piperacillin-tazobactam with MICs of 0.064, 8, 1, 0.5, and 6 g/mL, respectively, and was intermediately resistant to clindamycin with an MIC of 4 g/mL [3]. Susceptibility testing was performed by using the Etest (AB Biodisk-V, Solna, Sweden) and was interpreted in accordance with the guidelines of the Clinical and Laboratory Standards Institute [7]. Controversially, the case reported by Lopez *et al*. [5] showed that the isolate tested by Etest was susceptible to penicillin G, amoxicillin/clavulanate, piperacillin/tazobactam, imipenem, metronidazole, and clindamycin. The isolate reported here was susceptible to amoxicillin/clavulanate, rifampicin, imipenem, metronidazole, clindamycin, and vancomycin and resistant to penicillin G. Accordingly, a good outcome has been observed with an association of rifampicin and clindamycinin in the patient reported here.

**Table 1:** **Case reports of** ***Robinsoniella peoriensis*** **infections**

Patient	Sex/Age, years	Sample (delay after admission)	Identification	Underlining condition	Treatment	Community-acquired (CA) or health care-associated (HCA)	Outcome	Reference
Case 1	Female/61	Left-sided abdominal fluid collection	16 S rRNA gene sequencing	Laroscopic sigmoid colectomy due to diverticulitis	Percutaneous drainage vancomycin piperacillin-tazobactam (after unsuccessful treatment with moxifloxacin and clindamycin)	HCA	Improvement	[4]
Case 2	Female/68	Fluid from wound debridement	16 S rRNA gene sequencing	Open pelvic and femur fractures	Surgical wound debridement broad-spectrum antibiotics	CA	Improvement	[4]
Case 3	Male/45	Fluid from wound debridement(one month)	16 S rRNA gene sequencing	Open reduction and internal fixation of tibial and febular fractures	Wound debridement clindamycin linezolid (after unsuccessful treatment with clindamycin alone)	HCA	Improvement	[4]
Case 4	Female/79	Blood cultures (four days)	16 S rRNA gene sequencing	Coronary artery bypass surgery for myocardial infarction,diabetes, hypertension, and hypercholesterolemia	Piperacillin/tazobactam, levofloxacin, and metronidazole	HCA	Died	[4]
Case 5	Female/42	Blood cultures (five days)	16 S rRNA gene sequencing	Pancreatic cancer	Metronidazole	HCA	Died	[3]
Case 6	Male/50	Muscle hematoma punction	16 S rRNA gene sequencing	Alcoholic liver cirrhosis	Clindamycin and ciprofloxacin	HCA	Died	[5]
Case 7	Female/45	Necrotic tissue	16 S rRNA gene sequencing	Osteosynthesis for thoracolumbar scoliosis	Debridement of necrotic tissue and removal of the bone graft clindamycin and rifampicin	HCA	Improvement	Present report

rRNA, ribosomal ribonucleic acid.

## Conclusions

The risk for post-surgical infections and their health care-associated reliability depend on the surgical procedure, which is classified into three categories: clean, clean-contaminated, and contaminated-dirty procedure. Our patient benefited from a clean neurosurgical procedure, and *R. peoriensis* was recovered from surgical specimens concurrently with an enlarged distal wound dehiscence, leading to the diagnosis of a health care-associated infection. Among the seven previously reported human cases of *R. peoriensis* infection, only six had information about the history of infection. Except for one patient who developed *R. peoriensis* soft-tissue infection after open traumatic injury, in which the source of infection remained uncertain [4], and one patient presenting with an infected spontaneous muscular hematoma [4], all cases could be considered health care-associated infections (Table 1). Indeed, previous clinical isolates have been made from the following: the deep wound of the heel of a 79-year-old woman in Sweden without available information [2], one peripheral blood culture of a patient with pancreatic cancer five days after his admission [3], an intra-abdominal fluid collection complicating a laparoscopic colectomy in a 61-year-old woman [4], fluid effusion after wound debridement in a 68-year-old woman with open pelvic and femur fractures with gross soil contamination [4], intra-operative specimens one month after open reduction and internal fixation in a 45-year-old man with right tibial and fibular fractures [4], one blood culture four days after a coronary artery bypass surgery for myocardial infarction in a 79-year-old woman [4], and the punction of an infected spontaneous muscular hematoma in a 50-year-old patient with alcoholic liver cirrhosis [4].

The most common source of anaerobic infections in surgical patients is endogenous normal flora or environmental contamination of wound injuries [10]. Although this anaerobic bacillus has not yet been isolated from normal human flora, the presentation of a case of a post-surgical site infection following a sigmoid colectomy suggests that the source of infection was the disruption of the mucosal barrier [4]. Accordingly, *R*. *peoriensis* is known to colonize pig feces, indicating it as a colinic organism.

## Abbreviations

Bp, Base pair; MALDI-TOF-MS, Matrix-assisted laser desorption/ionization time-of-flight mass spectrometry; MIC, Minimal inhibitory concentration; PCR, Polymerase chain reaction; rRNA, Ribosomal ribonucleic acid.

## Consent

Written informed consent was obtained from the patient for publication of this manuscript and accompanying images. A copy of the written consent is available for review by the Editor-in-Chief of this journal.

## Competing interests

The authors declare that they have no competing interests.

## Authors’ contributions

J-MG took care of the patient and helped to analyze and interpret the patient data regarding the hematological disease and the transplant. NC and LL helped to analyze and interpret the patient data regarding the hematological disease and the transplant. AR helped to perform the microbiological investigations. MD helped to perform the microbiological investigations and was a major contributor in writing the manuscript. All authors read and approved the final manuscript.

## References

[B1] Cotta MA Whitehead TR Zeltwanger RL Isolation, characterization and comparison of bacteria from swine faeces and manure storage pits Environ Microbiol 2003 5 737 745 10.1046/j.1467-2920.2003.00467.x 12919409

[B2] Cotta MA Whitehead TR Falsen E Moore E Lawson PA Robinsoniella peoriensis gen. nov., sp. nov., isolated from a swine-manure storage pit and a human clinical source Int J Syst Evol Microbiol 2009 59 150 155 10.1099/ijs.0.65676-0 19126740

[B3] Shen D Chen R Ye L Luo Y Tang YW Robinsoniella peoriensis bacteremia in a patient with pancreatic cancer J Clin Microbiol 2010 48 3448 3450 10.1128/JCM.00477-10 20631102 PMC2937667

[B4] Gomez E Gustafson DR Colgrove R Ly T Santana R Rosenblatt JE Patel R Isolation of Robinsoniella peoriensis from four human specimens J Clin Microbiol 2011 49 458 460 10.1128/JCM.01305-10 21048018 PMC3020413

[B5] Lopez P Belda S Garcia M Royo G Infection of a spontaneous muscular hematoma due to Robinsoniella peoriensis, in a patient with alcoholic liver cirrhosis Enferm Infecc Microbiol Clin 2010 28 565 567 10.1016/j.eimc.2010.01.005 20627461

[B6] King HA Moe JH Bradford DS Winter RB The selection of fusion levels in thoracic idiopathic scoliosis J Bone Joint Surg Am 1983 65 1302 1313 6654943

[B7] Performance Standards for Antimicrobial Susceptibility Testing of Anaerobic Bacteria; Informational Supplement M11-S1 2009 Clinical and Laboratory Standards Institute, Chicago, IL18291689

[B8] Fenollar F Roux V Stein A Drancourt M Raoult D Analysis of 525 samples to determine the usefulness of PCR amplification and sequencing of the 16 S rRNA gene for diagnosis of bone and joint infections J Clin Microbiol 2006 44 1018 1028 10.1128/JCM.44.3.1018-1028.2006 16517890 PMC1393109

[B9] Fenollar F Levy PY Raoult D Usefulness of broad-range PCR for the diagnosis of osteoarticular infections Curr Opin Rheumatol 2008 20 463 470 10.1097/BOR.0b013e3283032030 18525362

[B10] Edmiston CE Krepel CJ Seabrook GR Jochimsen WG Anaerobic infections in the surgical patient: microbial etiology and therapy Clin Infect Dis 2002 35 S112 S118 10.1086/341931 12173119

